# Effective dissection for rectal cancer with lateral lymph node metastasis based on prognostic factors and recurrence type

**DOI:** 10.1007/s00384-021-03870-5

**Published:** 2021-02-01

**Authors:** Hajime Morohashi, Yoshiyuki Sakamoto, Takuya Miura, Daichi Ichinohe, Kotaro Umemura, Takanobu Akaishi, Kentaro Sato, Daisuke Kuwata, Keisuke Yamazaki, Taiichi Wakiya, Kenichi Hakamada

**Affiliations:** Department of Gastroenterological Surgery, Hirosaki University Graduate School of Medicine, Zaifu-cho 5, Hirosaki-shi, 036-8562 Japan

**Keywords:** Lateral lymph node dissection, Lateral lymph node metastasis, Prognostic factor, Rectal cancer, Total mesorectal excision

## Abstract

**Purpose:**

There are no reports showing the significance and effective range of dissection for patients with lateral lymph node metastasis (LLNM). This study aimed to investigate the indications for lateral lymph node dissection (LLND) in patients with LLNM based on prognostic factors and recurrence types.

**Methods:**

We reviewed 379 patients with advanced rectal cancer who were treated with total mesorectal excision plus LLND. We analyzed background factors and survival times of patients who had LLNM to determine prognostic factors and recurrence types.

**Results:**

Pathological LLNM occurred in 44 (11.6%). Among patients with LLNM, the predictors of poor prognoses, according to univariate analysis, were > 3 node metastases, the presence of node metastasis on both sides, and spreading beyond the internal iliac lymph nodes. Moreover, LLNM beyond the internal iliac region was found to be an independent prognostic risk factor. Twenty-eight of the 44 patients with lateral lymph node metastasis (64%) relapsed, 22 of whom had distant metastases and 11 of whom experienced local recurrences. Among the latter group, nine (20%) and two (5%) had recurrences in the central and lateral pelvis, respectively.

**Conclusion:**

The therapeutic benefit of resection was high, especially in patients with ≤ 3 positive lateral lymph nodes, one-sided bilateral lymph node areas, and positive nodes localized near the internal iliac artery.

## Introduction

For advanced rectal cancer, when the tumor is located below the peritoneal reflection, the incidence of lateral lymph node metastasis (LLNM) is 14–30%, and the prognosis for patients with LLNM is poor [1, 2]. Therefore, it is important to improve the treatment outcomes in cases of LLNM in order to anticipate better treatment results with lower rectal cancer, in general. Total mesorectal excision (TME) is used to treat lower rectal cancer in hospitals worldwide [3, 4]. Since advanced rectal cancer is known to have more local recurrence when treated only with TME, national guidelines suggest that various preoperative treatment strategies precede TME in order to reduce local recurrence [5]. In Europe and the USA, preoperative chemoradiotherapy (CRT) and TME are the standard treatment methods, whereas the conventional treatment methods in Japan are TME and lateral lymph node dissection (LLND) [6]. Local recurrence of rectal cancer includes recurrence due to an inadequate circumferential resection margin (CRM) and recurrence due to lateral lymph node involvement. Although local recurrence caused by inadequate CRM cannot be compensated using LLND, the technique has an advantageous preventive effect on lateral lymph node recurrence. Conversely, there is little evidence of the therapeutic effect of CRT on LLNM. According to a Japanese study, incidences of lymph node metastasis in 2916 patients with rectal cancer accounted for 20.1%. Among those who underwent LLND, however, the risk of pelvic recurrence was reduced by 50% and the 5-year survival rate was expected to improve by 8–9% [1]. Therefore, in high-volume centers in Japan, the recommended standard procedure for advanced low rectal cancer is TME plus central D3 resection and bilateral LLND.

In the JCOG0212 large-scale clinical trial that mainly targeted patients with clinically negative LLNM [7], the local recurrence rate in patients who underwent LLND was significantly lower than that of patients who did not undergo the procedure. LLND was particularly effective in suppressing local recurrence within the lateral pelvis, including the lateral lymph nodes [8]. Nonetheless, the results did not indicate that LLND improved overall survival. However, the therapeutic effect of LLND on patients with LLNM has not been clarified, and there is no prospective large-scale clinical trial showing this crucial data.

Previously, it has been reported that the dissection effect is high in cases with a small number of lateral lymph node metastases and in cases in which the metastases exist only in a limited area, but dissection does contribute to improved survival [9, 10]. However, there is no report showing the significance and effective range of dissection for LLNM. For these reasons, our study aimed to investigate the indications for LLND based on prognostic factors and recurrence type in patients with LLNM.

## Materials and methods

### Patients

**Fig. 1:**
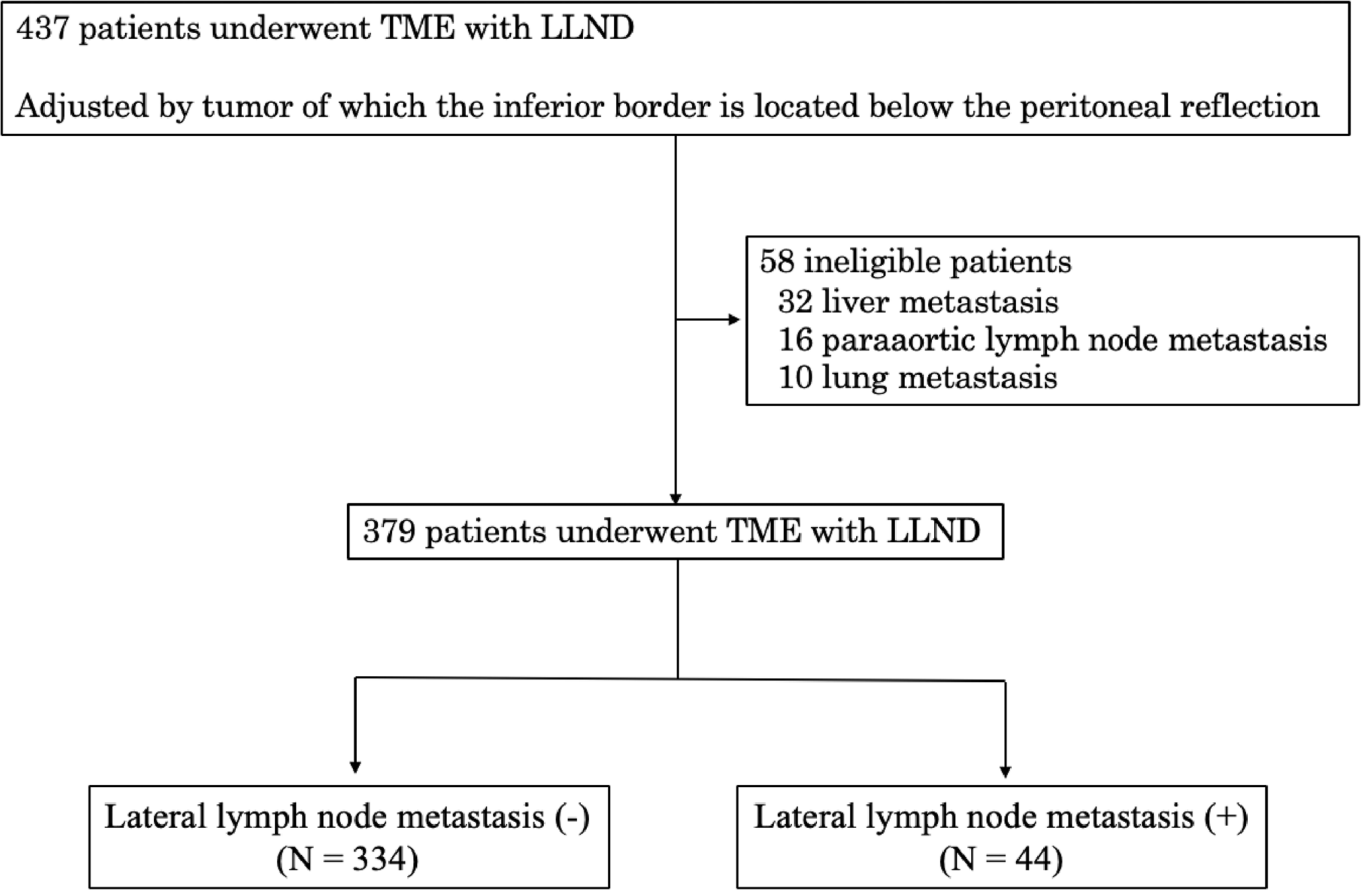
Patients’ profile

Between April 2001 and June 2017, 437 patients with low rectal cancer where tumor borders were below the peritoneal reflection underwent TME with LLND. The primary tumors were addressed pathologically with R0 resection at the Department of Gastroenterological Surgery, Hirosaki University. This study is from a single center, and the laboratory data or imaging data of all cases have been reviewed at a preoperative conference involving experienced surgeons. A total of 379 patients were included in our study after excluding 58 with distant metastases in the liver, lungs, or paraaortic lymph nodes (Fig. 1). The clinicopathological characteristics of patients were determined from clinical and histopathologic notes, and the tumor features and stages were classified according to the TNM classification system [11]. This retrospective study protocol was approved by the institutional Ethics Committee of Hirosaki University Hospital (No. 2018-120). Written informed consent was obtained from each patient prior to enrollment.

### Treatment strategies and surgical procedures

LLND was indicated when the lower margin of the tumor was located below the peritoneal reflection and the tumor invaded the serosa, such patients underwent TME with LLND without preoperative CRT. Tumor progression, size, and position were evaluated using diagnostic imaging such as multidetector-row computed tomography (CT), magnetic resonance imaging (MRI), and barium enemas. The indications were determined in accordance with the guidelines of the Japanese Society for Cancer of the Colon and Rectum [5]. All patients underwent bilateral LLND after TME; proximal lymph node dissection along the lower mesenteric artery was also performed. The location of the lateral resection was identified according to the classifications set forth by the Japanese Society for Cancer of the Colon and Rectum [12], and LLND was performed as previously described [13]. The location of the LLLD was the internal iliac lymph node area and the obturator lymph node area [1]. Internal iliac vascular region lymph nodes dissection involved the removal of the fatty tissue on both the ventral side of the internal iliac vein and internal iliac artery from the bifurcated cords of the umbilical artery to the lateral urinary bladder. Obturator lymph node dissection entailed removal of the fatty tissue from the dorsal side of the external iliac vein to the tendinous arch of the levator ani muscle along the internal obturator muscle. Local and distant metastasis was diagnosed by CT, MRI, positron emission tomography, and elevated tumor markers, not biopsy.

The indication of adjuvant chemotherapy was pStage III. In principle, S-1 or UFT/LV therapy was to be started within 8 weeks after surgery. However, depending on the postoperative stage, the addition of oxaliplatin or even omission of postoperative adjuvant chemotherapy was at the discretion of the attending physician.

### Statistical analysis

Survival analyses were performed using the Kaplan-Meier method with the log-rank test. Categorical variables are presented as patient percentages. *P*-values < 0.05 were considered statistically significant. All statistical analyses were performed using SPSS version 24 (IBM Inc., Armonk, NY).

## Results

### Clinicopathological details

The attributes of all 379 patients are shown in Table 1. The average age was 62 years (range: 24–80 years), and 70% were male. Lower rectal cancer (Rb) was diagnosed in 326 patients (86%), while the remaining subjects had upper rectal cancer (Ra). A pathologic wall depth of pT3 was found in 210 patients (55%). Pathological mesorectal lymph node metastasis was observed in 158 patients (42%) and pathological LLNM in 44 (11.6%). Neoadjuvant chemotherapy was administered to 58 patients; none received CRT. The indication of neoadjuvant chemotherapy was determined in the same manner as our new prospective study evaluating neoadjuvant chemotherapy without CRT for lower rectal cancer (unique trial no. jRCTs021180033). One hundred eighty-two patients (48.0%) were given adjuvant chemotherapy. Table 2 shows the rate of positive LLNM for pT1 (3.7%), pT2 (6.6%), pT3 (13.3%), and pT4 (15.2%).

**Table 1:** Patient characteristics

Characteristics	*n* = 379
Sex	
Male	264
Female	115
Age (year)	
Median	62
Range	24–80
Tumor location*	
Ra	53
Rb	326
Tumor size (mm)	
< 50	175
≧ 50	204
Clinical stage	
II	181
III	198
Histological type	
Well/moderate	76
Mucinous/poor/signet	303
Pathological T category	
pT1	27
pT2	76
pT3	210
pT4	66
Pathological mesorectal LN metastasis	
Absent	221
Present	158
Pathological lateral LN metastasis	
Absent	335
Present	44
Vascular invasion	
Absent	163
Present	216
Lymphatic invasion	
Absent	78
Present	301
Neoadjuvant chemotherapy	
No	321
Yes	58
Adjuvant chemotherapy	
No	197
Yes	182

*Ra, tumor located above the peritoneal reflection; *Rb*, tumor located the peritoneal reflection

**Table 2:** Rate of LLNM for pT1, pT2, pT3 and pT4

	Pathological T category	Lateral lymph node metastasis
	(*N* = 379)	(*N* = 44)
pT1	27	1 (3.7%)
pT2	76	5 (6.6%)
pT3	210	28 (13.3%)
pT4	66	10 (15.0%)

### Surgical outcomes

Patient surgical outcomes are summarized in Table 3. The mean operation time was 243 min, while the mean blood loss was 532 ml. Low anterior resection was the most common procedure (164 patients), and R0 excision was achieved for the primary lesion combined with proximal D3 node dissection in almost all patients. Thirty-eight patients underwent laparoscopic surgery, which tended to last longer than laparotomy, albeit with less bleeding. The median post-surgery hospitalization period was 26 days. In Japan, the length of hospital stay generally includes the period of medical treatment until food intake, defecation, and recovery of motor function after surgery, so the length of hospital stay tends to be longer than in Europe and the USA. Hospitalization can be significantly longer, especially when serious complications develop. Postoperative complications included anastomotic leakage in 57 (19%) of the 299 patients in whom low anterior resection plus intersphincteric resection was performed. Cases with large tumors and cases with NAC tended to have more anastomotic insufficiency and intra-abdominal abscesses.

**Table 3:** Operative outcomes

Variables	Value
Operative procedure	
Low anterior resection	164
Intersphincteric resection	135
Hartmann’s procedure	8
Abdonimoperitoneal resection	67
Total pelvic exenteration	5
Open or laparoscopic assisted	
Open surgery	341
Laparoscopic assisted	38
Operation time (min) (range)	243 (86–633)
Total blood loss (ml) (range)	532 (5–2500)
Postoperative hospital days (day) (rage)	26 (8–144)
Operative complication (cases)	
Anastomotic leakage	57 (19%)
Perineal wound infection	27 (33%)
Abdominal wound infection	17 (4.4%)
Paralytic ileus	18 (4.7%)
Bowel obstruction	20 (5.3%)
Urinary retention requiring self-catheterization at discharge	11 (2.6%)
Bleeding	3 (0.8%)
Pelvic abscess	26 (6.9%)

Perineal wound infections occurred in 27 (38%) of the 80 patients who underwent the combination of abdominoperineal resection and total pelvic exenteration. The onset of abdominal wound infection was noted in 17 patients (4.4%), ileus in 20 (5.3%), and pelvic abscess in 26 (6.9%). In addition to the patients in whom total pelvic exenteration was performed, 11 patients with dysuria (2.6%) required a urinary catheter at the time of hospital discharge.

### Prognosis

**Fig. 2:**
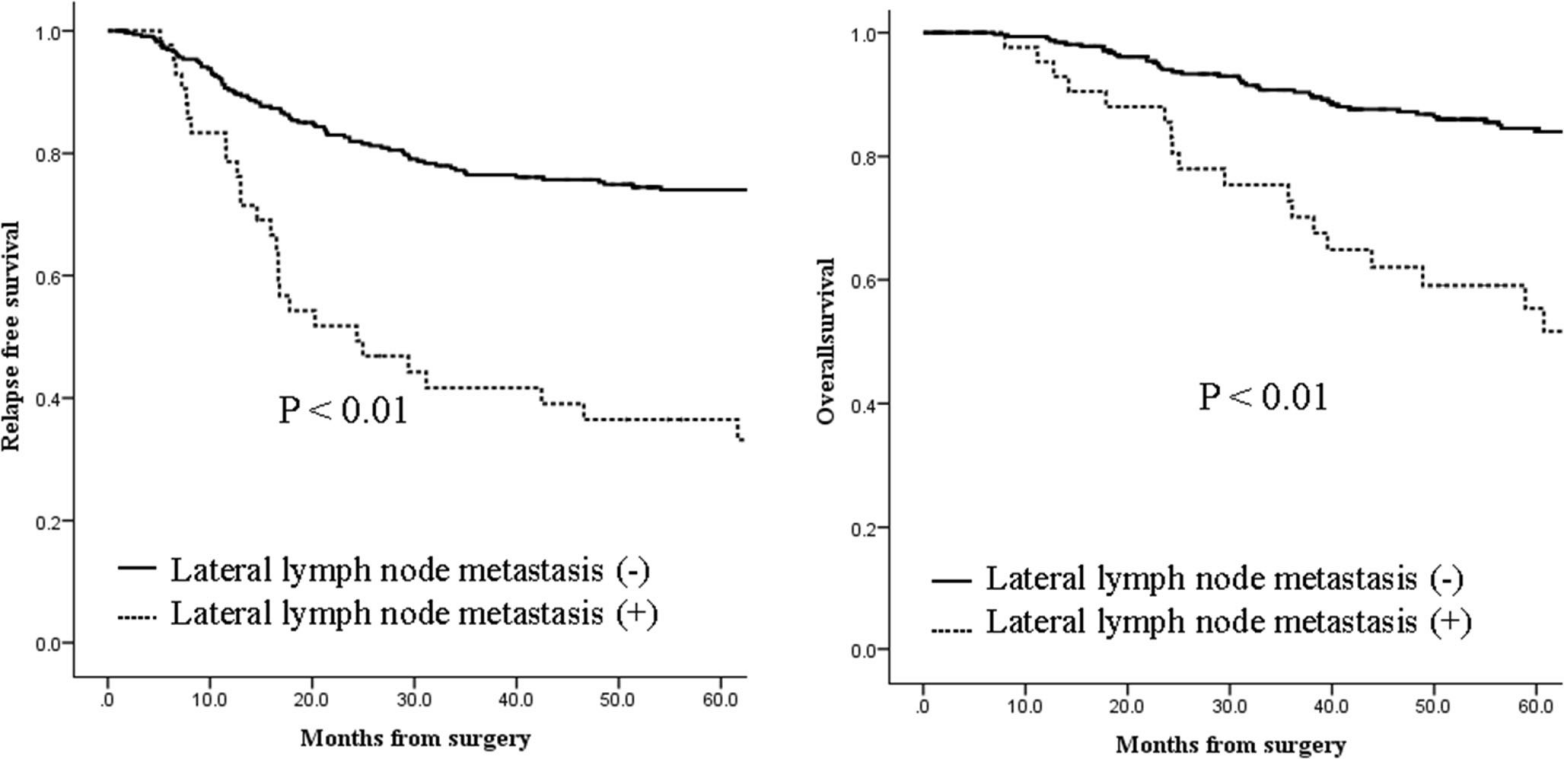
Relapse-free survival (**a**) and overall survival (**b**) rate of patients with and without lateral lymph node metastasis

The median observation period was 60.4 months (range: 36–96 months). Figure 2 shows relapse-free survival and overall survival. Among patients who underwent LLND, the 5-year survival rate was 55% and 85% for those with and without metastases, respectively (*P* < 0.01). Table 4 shows univariate and multivariate analysis of all patients who had TME and LLND. Among patients who underwent LLND, the predictors of poorer prognoses were identified as stage III disease, poorly differentiated tissue type, pT3–4, mesenteric lymph node metastasis, LLNM, venous invasion, or lymphatic invasion. On multivariate analysis, pT3–4 and lymphatic invasion were independent risk factors of poorer survival. Among patients with LLNM, the predictors of poor prognoses according to univariate analysis were > 3 node metastases, the presence of node metastasis on both sides, and spreading outside the internal iliac lymph node region. On multivariate analysis, LLNM outside the internal iliac and obturator lymph node area was found to be an independent prognostic risk factor (Table 5).

**Table 4:** Prognostic factors of lateral lymph node dissection

Variables	Univariate analysis			Multivariate analysis		
	HR	95% CI	*P* value	HR	95% CI	*P* value
Sex			0.793			
Male	1					
Female	0.934	0.558–1.562				
Age (year)			0.378			
≦ 60	1					
> 60	0.807	0.501–1.299				
Tumor location*			0.761			
Ra	1					
Rb	0.907	0.485–1.696				
Tumor size (mm)			0.799			
< 50	1					
≧ 50	1.065	0.658–1.722				
Clinical stage			0.001			0.065
II	1			1		
III	5.586	2.923–10.676		2.355	0.949–5.846	
Histological type			0.002			0.073
Well/moderate	1			1		
Mucinous/poor/signet	3.418	1.371–8.522		2.314	0.925–5.790	
Operative procedure			0.052			
Sphincter preservation/Hartmann	1					
APR/TPE	1.727	0.996–2.993				
pT			0.001			0.008
pT0-2	1			1		
pT3-4	6.324	2.302–17.371		3.98	1.440–11.003	
Pathological mesorectal LN metastasis			0.001			0.121
Absent	1			1		
Present	5.177	2.954–9.074		1.888	0.931–2.869	
Pathological lateral LN metastasis			0.001			0.087
Absent	1			1		
Present	3.145	1.834–5.394		1.635	0.931–2.869	
Vascular invasion			0.001			0.497
Absent	1			1		
Present	1.944	1.200–3.150		1.200	0.709–2.029	
Lymphatic invasion			0.001			0.022
Absent	1			1		
Present	2.575	1.599–4.149		1.843	1.092–3.111	
Neoadjuvant chemotherapy			0.760			
No	1					
Yes	1.140	0.492–2.640				

*Ra, tumor located above the peritoneal reflection; *Rb*, tumor located the peritoneal reflection

**Table 5:** Prognostic factors of lateral lymph node metastasis

Variables	Univariate analysis			Multivariate analysis		
	HR	95% CI	*P* value	HR	95% CI	*P* value
Sex			0.289			
Male	1					
Female	0.603	0.237–1.536				
Age (year)			0.662			
≦ 60	1					
> 60	1.223	0.495–3.021				
Tumor location*			0.571			
Ra	1					
Rb	21.418	0.001–8565.6				
Tumor size (mm)			0.808			
< 50	1					
≧ 50	1.12	0.449–2.792				
Clinical stage			0.343			
II	1					
III	2.655	0.354–19.935				
Histological type			0.830			
Well/moderate	1					
Mucinous/poor/signet	0.873	0.253–3.013				
Operative procedure			0.593			
Sphincter preservation/Hartmann	1					
APR/TPE	1.283	0.513–3.213				
pT			0.274			
pT0-2	1					
pT3-4	0.274	0.522–9.909				
Pathological mesorectal LN metastasis			0.269			
Absent	1					
Present	25.179	0.082–7722.9				
Pathological lateral LN metastasis			0.008			0.272
< 3	1			1		
≧ 3	0.308	0.129–0.732		1.548	0.710–3.377	
Pathological lateral LN metastasis			0.006			0.866
Unilateral	1			1		
Bilateral	3.258	1.401–7.575		1.068	0.499–2.284	
Pathological lateral LN metastasis			0.048			0.016
Internal iliac vascular region lymph nodes area	1			1		
Other obturator lymph node area	2.355	1.019–5.442		2.7	1.199–6.079	
Vascular invasion			0.365			
Absent	1					
Present	1.543	0.604–3.939				
Lymphatic invasion			0.537			
Absent	1					
Present	1.887	0.251–14.180				
Neoadjuvant chemotherapy			0.479			
No	1					
Yes	1.646	0.479–5.655				

*Ra, tumor located above the peritoneal reflection; *Rb*, tumor located the peritoneal reflection

### Recurrence

**Fig. 3:**
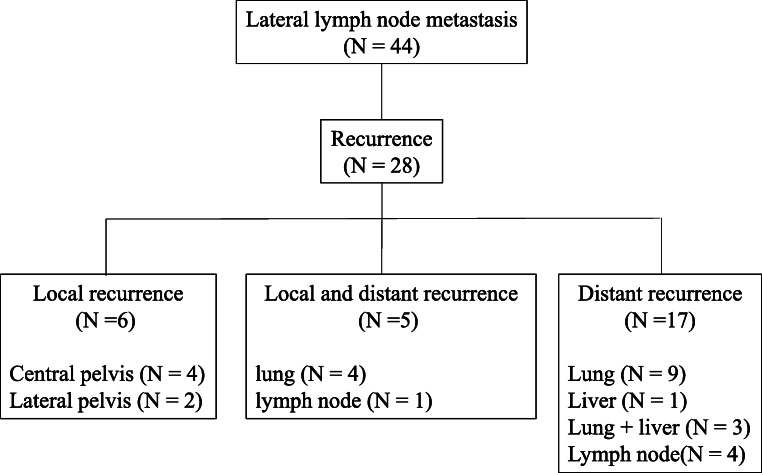
Recurrence rate of local and distant metastasis with lateral lymph node metastasis

**Fig. 4:**
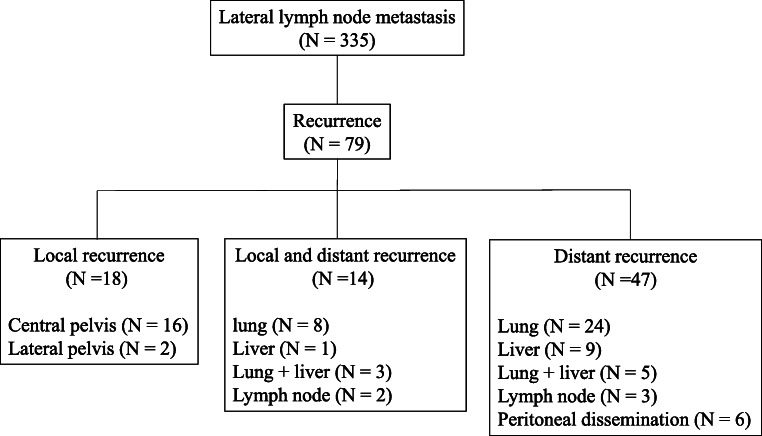
Recurrence rate of local and distant metastasis without lateral lymph node metastasis

Figure 3 shows a flowchart of recurrence in patients with LLNM. Twenty-eight of the 44 patients with LLNM (64%) relapsed; 11 experienced local recurrences (nine in the central pelvis and two in the lateral pelvis) while 22 (79%) had distant recurrences. Figure 4 shows a flowchart of recurrence in patients without LLNM. Seventy-nine of the 335 patients with LLNM (23.6%) relapsed; 32 experienced local recurrences (30 in the central pelvis and two in the lateral pelvis) while 61 (77.2%) had distant recurrences.

## Discussion

Examination of prognostic factors revealed that metastases of > 3 nodes, the presence of node metastasis on both sides, and spreading outside the internal iliac lymph node region led to a poor prognosis. LLNM outside the internal iliac and obturator lymph node area, in particular, was found to be an independent prognostic risk factor. The indication of LLND for patients with LLNM differs depending upon whether the LLNM is considered to consist of local metastases that can be cured by resection or distant metastases that cannot be controlled locally [14]. There are various opinions on whether and how bilateral LLND should be performed for LLNM [15], as there are other options, such as using selective LLND only for swollen lymph nodes [16] and omitting LLND, substituting CRT [17]. The therapeutic benefits of LLND for patients with LLNM have not yet been definitively determined.

One of the essential goals of treating rectal cancer is to reduce the local recurrence rate, and procedures to this effect are being optimized in various countries worldwide. In Japan, TME with autonomic nerve-sparing LLND has been performed for a long time [18, 19]. This differs from the therapeutic strategies employed in Europe and the USA, where the standard treatment methods are preoperative CRT followed by TME. The reason for this is that the local rectal lymphatic system below the inverted peritoneum flows in three directions: upward, laterally, and downward [20]. In addition to dissecting the regional and central lymph nodes along the inferior mesenteric artery, in Japan, it is thought that R0 resection is possible by dissecting the region along the lateral lymph flow [9].

LLND in patients with rectal cancer has been reported to reduce local recurrence rates and increase 5-year survival rates [21, 22]. Conversely, a meta-analysis of 20 patients indicated no improvement in prognosis following lateral LLND, although an increase in urogenital system complications was observed [23, 24]. However, many Japanese surgeons use autonomic nerve-preserving LLND techniques, which is more likely to prevent such complications [15]. Among patients enrolled in the JCOG0212 trial [8], there was no significant increase in the rate of urinary dysfunction for those who underwent LLND compared with other procedures. The incidence of urinary dysfunction is associated with tumor location and blood loss [25]. More recently, robot-assisted lateral resection has been reported to reduce urogenital system complications caused by this procedure [26].

On the other hand, preoperative CRT is reportedly effective in controlling local recurrence but does not necessarily improve the prognosis [27]. There are also reports of adverse events such as high incidences of urogenital system disorders, as well as radiation-induced venous thrombosis, intestinal obstruction, fistula development, transcervical fractures, and anal function failures [28, 29]. Akiyoshi et al. reported that 66% of patients who were diagnosed with metastasis of the lateral lymph nodes via preoperative imaging examinations and who underwent LLND after CRT were found not to be cancer-free pathologically [16]. It appears that CRT is not always sufficient to treat enlarged metastatic lateral lymph nodes, as it cannot completely eliminate lateral lymph node metastases. Hence, LLND is the most useful approach to achieving local control in patients with enlarged metastatic lateral lymph nodes, whereas preoperative CRT is not recommended [30].

In this study, among patients who underwent LLND with metastases, the 5-year survival rate was 55% and the rate of LLNM recurrence was 64%; among those with recurrences, 25% were local (5% in the lateral pelvis and 20% in the central pelvis) and 79% were distant. Although local control within the lateral lymph node region was reasonably achieved following LLND, it is also necessary to aim for high-quality TME to reduce the risk of recurrence in the central pelvis. R1 resection is one of the risk factors of local recurrence. However, in this study, the subjects were patients who had R0 resections. Of the 197 patients who did not undergo adjuvant chemotherapy, 24 (12.2%) relapsed. Since there is a risk of recurrence in cases with pStage II or lower, as well, it is necessary to consider the indication of adjuvant chemotherapy.

In general, the 5-year survival rate for patients with stage IV rectal cancer is 10–20% unless the metastatic tumors are excised [31, 32]. Our study showed that patients with lateral lymph node metastases had a better prognosis with LLND than that of stage IV patients. Kanemitsu et al. reported that, according to the index of the estimated benefits derived from LLND, dissection of the internal iliac and obturator nodes yielded similar therapeutic benefits as those expected from the dissection of the superior rectal artery lymph nodes [15]. Furthermore, Tamura et al. reported no oncological benefit from performing LLND in patients with stage IV disease who had both lateral and distant lymph node metastases [33]. Oh et al. reported that 59.1% of their patients with lateral lymph node metastases who underwent LLND also had distant metastases and poor prognoses [34]. LLND may be beneficial for patients with LLNM, but its indication is restricted if cancer cells have potentially spread to other areas of the body.

It is very difficult to determine the presence of LLNM before surgery. A previous study identified the risk factors for LLNM as female sex, lower rectal cancer location, lymphatic invasion, venous invasion, wall progression, and paraintestinal lymph node metastasis ≥ 5 mm in width on magnetic resonance imaging [35]. Another group found that the diagnostic ability for LLNM is more promising at a short axis of 5 mm than it is at 10 mm [36]. However, there are reports of pathological lymph node metastases being found in approximately half of the lymph nodes that were judged to be small (< 5 mm) on diagnostic imaging [37, 38]. It may be difficult to evaluate the presence or absence of LLNM based only on the size of preoperative imaging. Hence, it may be necessary to more closely evaluate the shape and integrity of the lymph nodes in addition to the size for a more thorough analysis of the presence of metastases. Our research group recently reported that a combination of size-based diagnoses and dual-energy CT may increase the accuracy of a preoperative diagnosis of LLNM [39].

When accurate preoperative determination of LLNM is difficult, but metastasis is suspected, LLND is an appropriate approach for lower rectal cancer. The therapeutic effect of resection is considered high, especially in patients with ≤ 3 positive lateral lymph nodes, nodes on one side of the bilateral lymph node area, or nodes localized near the internal iliac artery. Local control is achievable for patients with LLNM following LLND; however, distant recurrence is frequent in LLNM. Given the high proportion of distant recurrences, a strategy that considers potent systemic chemotherapy for distant metastases is necessary to improve the survival rate of patients with lateral lymph node metastases.

The limitation of our research is that it was a retrospective study, and the study sample of LLNM (*n* = 44), which we used for determining risk factors, was small. Moreover, owing to the long study period, the laparotomy/laparoscopy procedures, chemotherapy regimens, scopes of lymph node resection, and treatment durations have evolved over the course of the study and were therefore inconsistent across the entire cohort. In Japan, traditionally, the mesorectum is peeled away and excised, after which it is examined for lymph node metastasis. Then, the rectum is opened and formalin is used to determine the extent of cancer. Thus, in this study, it was difficult to make an accurate pathological evaluation of CRM or TME quality on resected specimens in the same manner as in Europe and North America.

## Conclusion

Based on the prognostic factors and the recurrence types of patients with LLNM, it was thought that LLND could control the metastasis limited to the internal iliac vessel region with less than three metastases on one side. It was considered necessary to treat LLNM as a systemic disease in cases in which metastasis spread further.
